# 

**Published:** 1882-01

**Authors:** 


Vol. XXXVI. JANUARY, 1882. No. 1.

S.G.O. INDEXED

519

THE

### Dental Register,

A MONTHLY JOURNAL,

DEVOTED TO THE INTERESTS OF THE PROFESSION.

EDITED BY J. TAFT, D. D. S.

PUBLISHED BY

SPENCER & CROCKER,

OHIO DENTAL DEPOT,

NOS. 117, 119, 121 WEST FIFTH STREET, CINCINNATI, OHIO.

*TERMS—$2. Per Annum, in Advance. Single Copies, 25 Cents.*

